# Family Income and Children’s Emotional Wellbeing: the Mediational Role of Parents’ Life Satisfaction and Emotional Wellbeing in China

**DOI:** 10.3390/ijerph17207573

**Published:** 2020-10-18

**Authors:** Di Qi, Yichao Wu

**Affiliations:** 1 Department of Sociology, School of Public Administration, Hohai University, Nanjing 210098, China; 2 Department of Economics, School of Economics and Management, Southeast University, Nanjing 211189, China; wuyichao513@126.com

**Keywords:** family income, children’s emotional wellbeing, parents’ life satisfaction

## Abstract

Utilizing the Chinese Family Panel Studies (CFPS) dataset and the structural equation model, this paper examines the direct and indirect influences of family income on children’s emotional wellbeing as a function of parents’ life satisfaction and parents’ emotional wellbeing. Firstly, the empirical analysis of this paper shows that family income exerts a positive influence on children’s emotional wellbeing outcomes, including depressed, hopeless, helpless, and meaningless feelings. Secondly, the findings show that family income is significantly associated with parents’ emotional wellbeing, through which children’s wellbeing is affected. The intergenerational emotional transmission mechanism is validated. The ability to control personal emotions is an important skill, related not only to personal health but also to children’s health and wellbeing. Furthermore, parents’ life satisfaction serves as the mediator between family income and parents’ emotional wellbeing. If parents are more satisfied with their own lives, they are less likely to experience emotional problems. Policy implications are discussed in the end.

## 1. Introduction

Recently, there has been an increasing number of reports in the media related to suicide and extreme behaviors by children who are left behind by parents in rural China. For example, in 2015, four children committed suicide by drinking poison in Bijie in the Guizhou province, hurting many people. These events attracted wide public attention regarding the healthy development of children’s psychological or emotional health. Subsequently, children’s emotional wellbeing was highlighted by the central government. In 2016, the State Council of China issued notices on strengthening the care and protection for rural left-behind children, which stress the importance of strengthening the protection for left-behind children in rural areas of China, as they are more likely to be affected by psychological or emotional problems due to a lack of adequate care and the absence of parents (State Council, 2016) [1] Children’s emotional wellbeing is not only related to their health, but also correlates with their future development. For example, the study by Elgar et al. (2016) [2] demonstrated that income inequality experienced by female adolescents in early childhood affected their future health and wellbeing using pooled, multilevel data from the Health Behavior in School-Aged Children Study. In this context, children’s emotional wellbeing and its influencing factors need to be examined.

Children’s wellbeing involves their development in multiple domains, which not only refer to the promotion of children’s lives in the aspect of material living conditions, but also their subjective wellbeing and emotional development (Bradshaw, 2006; EU, 2008; Lau & Bradshaw, 2010; Lau & Li, 2011; Minujin & Nandy, 2012; UNICEF, 2013 [3,4,5,6,7,8]). Camfield et al. (2008 [9]) systematically reviewed previous studies in terms of the definition and measurement of children’s wellbeing, and they summarized previous measurements of children’s wellbeing, including objective and subjective approaches. Objective approaches measure wellbeing using externally verifiable indicators, such as children’s cognitive development outcomes represented by the Organization for Economic Cooperation and Development (OECD) Program for International Student Assessment, evaluating children’s multiple deprivations through a multidimensional poverty or deprivation index. A large number of existing studies analyzed children’s objective wellbeing status (Chzhen & de Neubourg, 2014; Chzhen et al., 2014; de Millliano & Plavgo, 2014; de Neubourg et al., 2012a, 2012b; Gordon & Nandy, 2012; Notten & Roelen, 2011; Qi & Wu, 2014, 2015; UNICEF, 2011 [10,11,12,13,14,15,16,17,18,19]). On the other hand, subjective measures of children’s wellbeing mainly refer to their subjective feelings, emotional responses, or life satisfaction (Diener, 2006 [20]).

Despite the importance of children’s emotional wellbeing, there is little evidence demonstrating the relationship between family income and children’s emotional wellbeing in China. The mediation mechanism is also not well explained. This paper examines the mediational role of parents’ life satisfaction and emotional wellbeing. The following questions are addressed: (1) What is the relationship between family income and children’s emotional wellbeing? (2) To what extent do parents’ life satisfaction and emotional wellbeing play a mediational role? (3) To what extent does family income explain children’s emotional wellbeing, controlling for mediators?

## 2. Literature Review

Some studies argued that a family’s limited financial resources may generate adverse impacts on children’s development outcomes (Boyden and Dercon, 2012; Duncan and Brooks-Gunn, 1997; Duncan et al., 1998; Duncan et al., 1994; Elgar et al., 2016; Fotso et al., 2012; Hannum et al., 2014; [21,22,23,24,25,26]), as they restrict parental investment in children’s development and wellbeing. In the emotional domain, access to financial resources was found to influence children’s emotional outcomes (Kendall & Li, 2005 [27]. Children from families with lower income are more likely to be depressed (Brooks-Gunn & Duncan, 1997; Patterson et al., 1989 [28,29]). They have more symptoms of psychiatric disturbance, as well as an increased likelihood of depression and maladaptive social functioning, compared with children from more affluent households.

The above literature mainly focused on the relationship between family income and children’s emotional wellbeing without examining the mediation mechanism. According to other research, parental emotional wellbeing may play this role. According to the family stress model, financial distress may exert an influence on parents’ psychological state, which then affects children (Conger et al., 2000, 2002 [30,31]). Through this process, parental mental health and emotional wellbeing affect children’s emotional status when parents feel distressed, and when they experience stressful life events and low-quality partner relationships (Horwitz et al. 2007 [32]). Some studies specifically focused on mothers and showed that maternal depression is associated with less sensitive and responsive interactions between mothers and children, as well as more negative emotional expression and unpredictable and inconsistent parenting techniques (Beck, 1996; Brennan et al., 2000, 2002, 2003; Campbell et al., 2004; Fergusson, 1995; Halligan et al., 2007; Lim et al., 2008; Stewart, 2007 [33,34,35,36,37,38,39,40,41]). Talge et al. (2007 [42]) found that mothers who are anxious during pregnancy are more likely to show fearful or anxious behavior themselves, which poses greater risk for children’s development. O’Donnell et al. (2014 [43]) found that mothers’ anxiety and depression during pregnancy predict children’s emotional problems, whereby children are more likely to suffer from mental disorders. In addition to mothers, fathers’ psychological distress was also found to be associated with children’s emotional problems (Fletcher et al., 2011; Kvalevaag et al., 2013 [44,45]). Research showed that parental depression significantly reduces positive parental behaviors such as warmth and increases negative parenting behaviors such as criticism (Wilson and Durbin, 2010 [46]). Quinn et al. (2014 [47]) demonstrated that a family’s social support and stress levels affect children’s mental health and wellbeing through the mediational effects of caregivers’ mental and physical health, as both affect the quality of caregiving practices. Overall, the intergenerational transmission of emotional problems hurts children.

Furthermore, some evidence showed that wealth is positively related to an individual’s life satisfaction (Diener et al., 2010 [48]). A higher level of income allows individuals to more easily fulfill their needs for food, housing, and services. It is also easier for rich individuals to achieve goals and fulfill desires; therefore, their personal happiness and life satisfaction are higher. The study by Plouffe and Tremblay (2017 [49]) showed a positive relationship between an individual’s income and their life satisfaction. With respect to families, parents may have lower life satisfaction due to restricted household income, which influences their own emotional health and subsequently their children’s wellbeing. The influence of the two mediators of parents’ life satisfaction and parents’ emotional wellbeing on children’s emotion should be further investigated, as no previous studies evaluated these mechanisms.

Furthermore, after a comprehensive review, most studies were found to be based in a Western context, whereas research focusing on Chinese children is scarce. Some studies examined adults’ wellbeing; for example, Lin (2016 [50]) analyzed adults’ subjective wellbeing and happiness using survey data collected in three cities (Hangzhou, Xiamen, and Shenzhen) during 2011 and 2012. Research by Ren and Treiman (2016 [51]) studied the emotional wellbeing of children in China but mainly investigated the influence of parental migration. As described in Section 1, despite the importance of children’s emotional health and wellbeing for their current and future development, very few studies empirically analyzed how family income is related to children’s emotional wellbeing in the context of China, and to what extent do parental life satisfaction and emotional wellbeing play a mediational role. This was the focus of this paper and its contribution to existing knowledge.

## 3. Methods

Empirical methods were designed to investigate the relationship between family income and children’s emotional wellbeing. On the basis of the discussion of previous literature, this study proposes the following hypothesis: a higher family income results in better children’s emotional wellbeing. In order to test this proposed hypothesis and to empirically estimate the relationship, this paper firstly used the multivariate regression method, with the model specification shown below.

(1) $$Y_{i}=\alpha +\beta *X_{i}+\gamma *Z_{i}+\mu_{s}+\lambda_{t}+\varepsilon$$

where *Y_i_* denotes the dependent variable, i.e., the emotional wellbeing of child *i*, *X_i_* indicates the independent variable of interest, i.e., the family income of child *i*, and *β* represents the coefficient of interest that interprets their causal relationship. In addition, this model controls for a vector of children’s individual characteristics, *Z_i_*. Furthermore, *μ_s_* and *λ_t_* are the fixed effects of province and year respectively.

However, the mechanism behind this relationship remains a black box, and the influence of income may not be straightforwardly linked to children’s emotion, especially for young children. According to the conclusions and findings of previous research, parents’ own emotional wellbeing may impact children’s emotional wellbeing, i.e., intergenerational transmission of emotional wellbeing; thus, it may play a mediational role in the connection between both ends of the hypothetical relationship. Therefore, this paper further proposes the following hypothesis to illustrate the mediation mechanism: a higher family income is linked to better parents’ emotional wellbeing, which then translates to better children’s emotional wellbeing.

**Figure 1:**
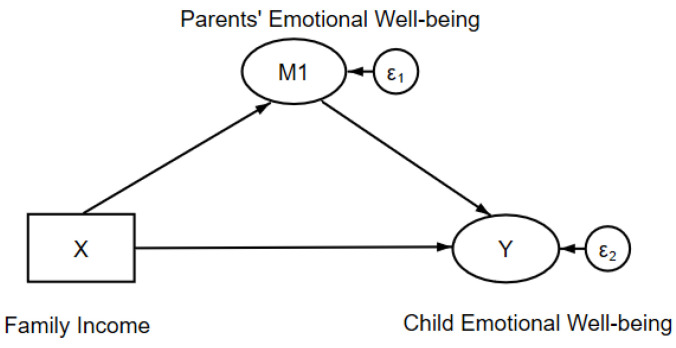
Conceptual model of the mediation effect of parents’ emotional wellbeing.

In order to test this proposed hypothesis and to empirically explain the mediation effect of parents’ emotional wellbeing, this paper next employed a structural equation model with the graphical illustration shown in Figure 1. There were two paths of the impact of family income (X) on children’s emotional wellbeing (Y): a direct effect and an indirect effect through parents’ emotional wellbeing (M1). A structured estimation of these two effects was the research object, and the significance of these coefficients allowed testing the above hypothesis.

Furthermore, although this mediation mechanism may help explain the causal relationship between family income and children’s emotional wellbeing to some extent, a higher family income may not necessarily lead to better parents’ emotional wellbeing; on the contrary, it may lead to upset and nervous feelings. One of the innovative contributions of this paper to the academic field is the establishment of a linkage via another mediator. Previous studies found wealth to significantly benefit life satisfaction, which may be highly related to a healthier emotional status. Therefore, this study employed parents’ life satisfaction as the mediator of the connection between family income and parents’ emotional wellbeing, finally proposing the following hypothesis: a higher family income is linked to higher parents’ life satisfaction, which leads to better parents’ emotional wellbeing, and, subsequently, better children’s emotional wellbeing.

**Figure 2:**
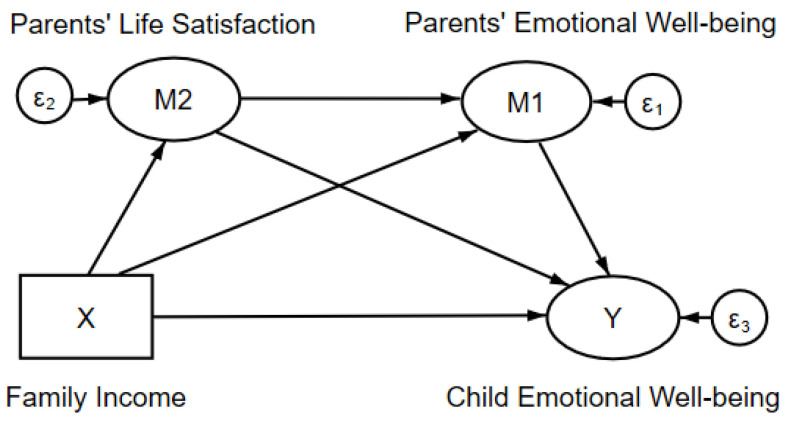
Conceptual model of the mediation effect of parents’ life satisfaction and emotional wellbeing.

Finally, in order to test this proposed hypothesis and to clarify the mediation effects, the empirical estimation was once again established using a structural equation model, but with a more complicated relationship, as shown in Figure 2. According to this graph, the influence of family income includes four paths in total: one direct effect and three indirect effects, through parents’ life satisfaction (M2), through parents’ emotional wellbeing (M1), and through parents’ life satisfaction and then emotional wellbeing. A structured estimation of these paths was the research object, and the significance of these coefficients then allowed an indication of which path truly exists.

## 4. Data

This study used Chinese Family Panel Studies (CFPS) as the main data source for the empirical analysis. The Institute of Social Science Survey at Peking University conducted the base survey of CFPS in 2010. The survey was originally designed to collect the socioeconomic information of Chinese families and individuals, as well as children’s characteristics such as health, education, and wellbeing. The CFPS investigation was carried out with a stratified sampling method including three levels: counties, communities, and households; each household was then selected according to the probability proportion to size (PPS). The main strength of this database is its national representativeness, as it investigated 25 provinces of China. Thus, this study used CFPS datasets in the wave of 2014, with a sample of 8616 children aged from 0 to 15 years old, to examine how children’s family income affects their emotional wellbeing. All types of households were covered in the sample, such as two-parent and one-parent families; thus, the data are adequately representative of Chinese households.

The dependent variable of this study was children’s emotional wellbeing, containing six specific indicators from the CFPS dataset. Table 1 lists the variables used in this paper and their corresponding descriptions in the questionnaire. All questions were presented using a five-point Likert scale ranging from 1 (“nearly every day”) to 5 (“never”), reflecting the frequency of children’s emotional problems, whereby a higher value denotes better children’s emotional wellbeing. However, due to the understanding required for these psychological questions, only children of age greater than 10 years old were surveyed with respect to these questions in 2014; therefore, the effective sample size contained 2567 children.

The independent variable of interest was family income or, more specifically, the household’s annual income per person, reflecting the family’s economic and material living conditions; this study took the natural logarithm of income. In addition, three control variables were used to further depict children’s individual characteristics: age, gender, and urban/rural residence.

Moreover, in terms of the two mediators, the indicators of parents’ emotional wellbeing used the exact same variables as for children, with fathers and mothers questioned separately (M101–M106) to reflect their frequency of suffering each emotional problem on a scale from 1 (“nearly every day”) to 5 (“never”). Secondly, the indicators of parents’ life satisfaction contained three dimensions: the satisfaction degrees of their interpersonal relationship, marriage, and life, with each dimension consisting of three individual questions, whereby fathers and mothers were questioned separately (M201–M209). Table 2 lists all corresponding descriptions in the questionnaire. Specifically, the satisfaction degree of their interpersonal relationship (i.e., the first three questions) was measured on a 10-point scale, while the satisfaction degrees of marriage and life (i.e., the remaining six questions) were measured on five-point scales. For all nine indicators, a higher value indicated a higher degree of life satisfaction.

## 5. Results

This study aimed to examine the relationship between family income and children’s emotional wellbeing, and Table 3 displays the empirical regression results which demonstrate the direct effect of family income, controlling for children’s individual characteristics (i.e., age, gender, and residence area). According to the results, family income exerted a positive and significant influence on four of six indicators of children’s emotional wellbeing. As household income increased, children’s frequency of suffering depressed, hopeless, helpless, and meaningless emotions significantly decreased, thus significantly benefiting children’s emotional wellbeing. The other two coefficients related to nervous and restless emotions were also positive, albeit not significant. These results highlight the importance of the family’s economic and material living conditions for children’s emotional wellbeing, empirically verifying the first hypothesis.

Furthermore, the individual characteristics of children also affected their emotional wellbeing. Firstly, as shown in Table 3, with an increase in age, children’s frequency of suffering all six emotions decreased, with a significantly lower frequency of older children feeling that life was meaningless compared to younger children. Secondly, boys showed a significantly higher frequency of feeling restless and meaningless than girls, which indicates that girls are more mature with respect to such emotional issues than boys, while there was no obvious gender difference for other indicators. Thirdly, living in an urban area significantly benefited children’s emotional wellbeing, especially in terms of feeling helpless and meaningless, thereby reflecting the influence of residence environment to some extent.

**Figure 3:**
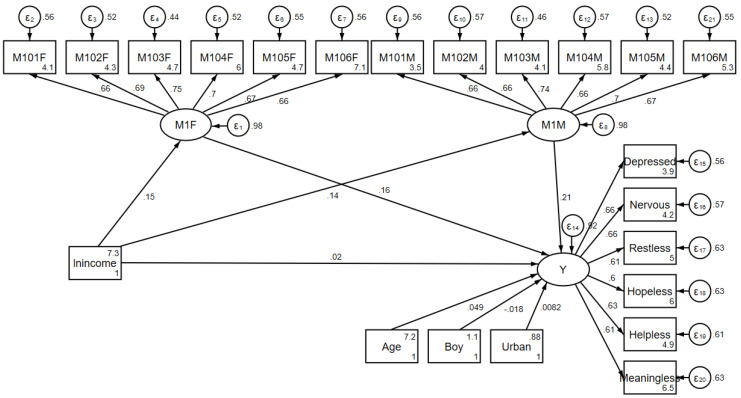
Structural equation model describing the mediation of parents’ emotional wellbeing.

As discussed above, in order to describe the impact mechanism of family income on children’s emotional wellbeing, the reduced form of the regression model was not enough. Hence, to test the hypothesis of certain mediation effects, this paper employed a structural equation model to estimate the direct and indirect influences. The structural equation model allows successfully dealing with a certain number of latent and observed variables/indicators. Figure 3 depicts the structure in more detail, with standardized coefficients shown alongside the arrows. According to this structure, the independent variable was still family income, while the six dependent variables, indicating children’s emotional wellbeing, were grouped as one latent dependent variable. More importantly, this structure introduced the latent mediator of parents’ emotional wellbeing (M1) for fathers and mothers, measured using the same six indicators for children’s emotional issues (M101–M106), resulting in 12 indicators in total. Lastly, the three children’s individual characteristics (age, gender, and residence) were also controlled for in this model.

According to the standardized coefficients in Figure 3, the direct effect of family income on children’s emotional wellbeing was about 0.02, but not significant, whereas the indirect effect through fathers’ emotional wellbeing was 0.024 (0.15 × 0.16) and that through mothers’ emotional wellbeing was 0.029 (0.14 × 0.21), with both estimates significant at the 99% confidence level. These results indicate that there was no significant direct influence of family income on children’s emotional wellbeing, whereas the positive effect of family income could only be explained by the mediation effect of parents’ emotional wellbeing, which verifies the second hypothesis proposed above, i.e., the intergenerational transmission of emotional wellbeing. In addition, age as one of the three children’s individual characteristics significantly affected children’s emotional wellbeing, whereby older children suffered significantly fewer emotional issues than younger children, whereas the other two control variables were not significant. Moreover, according to the measurement model and the corresponding standardized coefficients in Figure 3, the indicators selected were significantly and consistently correlated with the latent mediator and the latent dependent variable, respectively.

**Figure 4:**
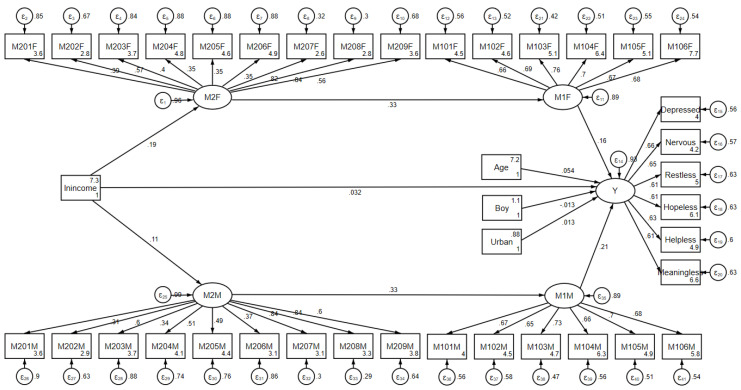
Structural equation model describing the mediation of parents’ life satisfaction and emotional wellbeing.

Furthermore, as discussed above, the indirect effect of family income on children’s emotional wellbeing through parents’ emotional wellbeing may be incomplete, and an additional mediator may be required to link family income to parents’ emotional wellbeing. Hence, this study added the mediator of parents’ life satisfaction to complete the linkage, as illustrated in Figure 4, with standardized coefficients shown alongside the arrows. Building on the previous structural equation model, the new latent mediator of parents’ life satisfaction (M2) was measured using 18 specific indicators, separately for fathers and mothers (M201–M209). The introduction of the new mediator split the indirect impact mechanism underlying the relationship between family income and children’s emotional wellbeing into three paths: through parents’ life satisfaction, through parents’ emotional wellbeing, and through parents’ life satisfaction and then emotional wellbeing.

As shown in Figure 4, the standardized direct effect of family income on children’s emotional wellbeing was about 0.022, but not significant. On the other hand, the standardized indirect effect showed that the effect of family income on children’s emotional wellbeing through fathers’ life satisfaction and emotional wellbeing was 0.01 (0.19 × 0.33 × 0.16) and that through mothers’ life satisfaction and emotional wellbeing was 0.007 (0.11 × 0.33 × 0.21). These results indicate that there was no significant direct influence of family income on children’s emotional wellbeing, whereas the positive effect of family income could only be explained via the mediation effect. Specifically, the indirect effect through parents’ emotional wellbeing was still significant, but with a smaller effect size than in the previous model, again verifying the second hypothesis, i.e., the intergenerational transmission of emotional wellbeing. In addition, the indirect effect through parents’ life satisfaction and then emotional wellbeing further explained the cause of intergenerational transmission of emotional wellbeing, thereby completing the linkage and verifying the third hypothesis. Similarly, age significantly affected children’s emotional wellbeing, with older children suffering significantly fewer emotional issues than younger children, whereas the other two control variables were not significant. Moreover, on the basis of the measurement model and the corresponding coefficients in Figure 4, the indicators selected were significantly and consistently correlated with the two latent mediators and the latent dependent variable, respectively.

## 6. Conclusions

Wellbeing refers to the realization of children’s rights and the fulfilment of an opportunity for every child to realize their ability and potential (Bradshaw, et al., 2006). Monitoring and measuring children’s wellbeing status is critical for better promoting children’s basic rights, as ratified in the United Nations Convention on Children’s Rights (UNCRC, 1989 [52]). As regulated in Article 17, all state members should ensure that all children have “access to information and material from a diversity of national and international sources, especially those aimed at the promotion of his or her social, spiritual, and moral wellbeing and physical and mental health” (UNCRC, 1989). The United Nations International Children’s Emergency Fund (UNICEF) Innocenti Research Center has also published a series of studies measuring the life qualities and wellbeing of children in economically developed countries (UNICEF, 2007 [53], 2013). For example, Innocenti Report Card 7 compared the status of children’s wellbeing among OECD countries in six dimensions encompassing material wellbeing, children’s health and safety, educational wellbeing, family and peer relationships, behaviors and risks, and subjective wellbeing. Three indicators were used for assessing the psychosocial aspects of subjective wellbeing, namely, the measurement of feelings of awkwardness (“I feel awkward and out of place”), loneliness (“I feel lonely”), and being an outsider (“I feel like an outsider or left out of things”) (UNICEF, 2007).

Other psychological scales have also been developed and applied internationally in different countries for assessing children’s subjective wellbeing or psychological health in both a positive and a negative way. For example, the Brief Multidimensional Students’ Life Satisfaction Scale (BMSLSS) (Seligson et al., 2003; Abedi and Vostanis, 2010 [1], Siyez and Kaya, 2007 [52,54,55,56]) with a seven-point response scale ranging from 1 (terrible) to 7 (delighted) was developed for assessing children’s life satisfaction toward family life, friends, school, and living environment. Similar scales include the Students’ Life Satisfaction Scale (Huebner, 1991 [57]), Multidimensional Students’ Life Satisfaction Scale (Huebner, 1994 [58]), Perceived Life Satisfaction Scale (Adelman, et al., 1989 [59]), and Elementary School Students’ Subjective Wellbeing in School Scale. The Strengths and Difficulties Questionnaire (SDQ) encompasses questions assessing both the positive and the negative psychological wellbeing of children. It contains 25 items assessing children’s emotional symptoms, conduct problems, hyperactivity, peer relationship problems, and prosocial behavior (Goodman, 1997 [60]).

As discussed in the beginning of this paper, emotional wellbeing is very important for children’s current and future development. It should cover indicators reflective of their feelings, subjective satisfaction status, emotional problems, and so on. Six indicators were selected in this paper describing their feelings of depression, nervousness, restlessness, hopelessness, helplessness, and meaninglessness. These six indicators adequately reflect children’s subjective feelings, psychological fluctuations, autonomy, and negative attitudes toward life. A good emotional status is beneficial for children’s physical and psychological health. As limited by the dataset, behavioral problems were not included. The six subjective questions were selected to reflect children’s wellbeing, overwhelmingly encompassing their emotions. The rich information of children’s emotional wellbeing is a good expansion of existing studies related to this measurement. 

The empirical analysis of this paper showed that family income exerted a positive influence on children’s emotional wellbeing outcomes including depressed, hopeless, helpless, and meaningless feelings. Children of lower-income families suffered more frequently from emotional problems. This indicates the necessity to protect children’s emotional wellbeing and psychological health in disadvantaged families. Furthermore, the results showed that age was a protective factor. Older children suffered fewer emotional issues, having experienced more positive and negative life events, having had more interactions with parents in dealing with difficulties, and having gained more knowledge and information from their family, school, or community. All these experiences help with overcoming emotional problems. Girls were found to experience fewer emotional troubles, potentially due to a warmer parental interaction and their greater maturity.

The contribution of this paper was to examine the direct and indirect influence of family income on children’s emotional wellbeing through parents’ life satisfaction and parents’ emotional wellbeing. Firstly, family income was found to be significantly associated with parents’ emotional wellbeing, through which children’s wellbeing was affected. In low-income families, parents suffer a greater risk of experiencing negative life events, such as unemployment, divorce, separation, and poor family relationships. These negative life experiences and challenges pose parents at greater risks of experiencing emotional disorders such as sadness, depression, hopelessness, nervousness, helplessness, and meaninglessness. These negative emotions are then transmitted to their children. Parents with negative emotions are more likely to display them when talking and interacting with children. This may result in less warmth during interactions, less encouragement, and fewer smiles, but harsher criticism, shouting, scolding, and beating, thereby creating an intergenerational emotional transmission mechanism. A higher likelihood of parents’ negative emotions results in a higher likelihood of children’s negative emotions. Previous studies discussed the intergenerational transmission of poverty (Boyden and Dercon, 2012 [7]; Duncan and Brooks-Gunn, 1997 [22]) through education or similar mechanisms; however, more interestingly, the emotional transmission between parents and children offers another theoretical explanation for poverty or social reproduction. The ability to control personal emotions is an important skill, which is related not only to personal health but also to children’s health and wellbeing. To achieve children’s development goals, policymakers and practitioners should highlight parents’ emotional status. Relevant courses should be provided to improve the emotional management skills of parents and children.

Furthermore, through the structural equation model, the analysis showed a significant association between family income and parents’ life satisfaction. Parents’ life satisfaction served as a mediator between family income and parents’ emotional wellbeing. Parents’ emotional wellbeing was one of the significant mediators linking family income, parents’ life satisfaction, and children’s emotional wellbeing. Income is related to individuals’ happiness, interpersonal relationships, and satisfaction with their marriage and life. People with higher income can more easily access a series of goods and services, such as better housing, nutrition, hospitals, community, and living environment. The ownership of better materials and resources results in individuals being satisfied with their life and having more confidence in their future. This satisfaction toward life serves as a protective factor in parents’ emotional wellbeing due to the presence of more emotional resources and capital to overcome life’s challenges and difficulties, e.g., good marriage partners, good colleagues, and good family members. With more confidence, they are more able to experience better and more stable emotions. Life satisfaction, thus, influences parents’ emotional wellbeing, subsequently impacting children’s emotions. Income only becomes a significant factor for children’s emotional wellbeing when included in a structural model incorporating the effects of parents’ life satisfaction and emotional wellbeing. This demonstrates the importance of parents’ subjective feelings and emotions for children’s health.

Moreover, as a function of the empirical findings, this study proposes some policy implications. Firstly, parents’ emotional status is essential for children’s emotional wellbeing, validating the intergenerational transmission of emotions. Therefore, relevant courses including how to manage emotions, how to improve personal autonomy toward life, and how to reduce the negative effects of emotional problems should be delivered at the community level for families with children. Secondly, parents’ life satisfaction is important for their emotional health. Parents are encouraged to take courses with the aim of improving life satisfaction and happiness. Thirdly, family income becomes a protective factor for children’s emotional wellbeing through the mediators of parents’ life satisfaction and emotional wellbeing. Children from lower-income families face an increased risk of experiencing emotional issues. Relevant resources should be provided to lower-income families; for example, social workers can link relevant courses for lower-income parents and children to improve their emotional wellbeing.

Lastly, when reviewing the existing policies on children in China, it can be found that the State Council of China has promulgated three National Child Development Plans and Outlines since the 1990s. The three children’s development plans targeted an improvement in children’s development and wellbeing, primarily in the domains of health, education, protection, and the environment. Despite the wide coverage of these outlines, the monitoring indicators of children’s development and wellbeing mainly focused on objective wellbeing indicators such as malnutrition or enrolment in schools rather than subjective indicators. In the future, more subjective or emotional wellbeing indicators should be included. This also applies to the children’s wellbeing monitoring system, whereby the National Working Committee on Women and Children under the State Council should highlight children’s emotional wellbeing indicators. In the introduction, we highlighted that left-behind children’s emotional problems have been highlighted due to suicide events in rural China (State Council, 2016). Beyond this group, a wider coverage of lower-income families and children is necessary.

## Figures and Tables

**Table 1:** Indicators of children’s emotional wellbeing.

Variable	Questions
Depressed	How often in the past month did you feel depressed?
Nervous	How often in the past month did you feel nervous?
Restless	How often in the past month did you feel restless?
Hopeless	How often in the past month did you feel hopeless about the future?
Helpless	How often in the past month did you feel it was difficult to do anything?
Meaningless	How often in the past month did you feel that life was meaningless?

**Table 2:** Indicators of parents’ life satisfaction.

Variable	Questions
M201	How is your interpersonal relationship?
M202	How happy are you?
M203	How good are you at getting along with others?
M204	Satisfaction with your marriage
M205	Satisfaction with your partner’s contribution to family income
M206	Satisfaction with your partner’s contribution to housework
M207	Satisfaction with your own life
M208	Satisfaction with your family life
M209	Confidence in your future life

**Table 3:** Regression results of children’s emotional wellbeing as a function of family income.

Variables	Depressed	Nervous	Restless	Hopeless	Helpless	Meaningless
Ln income	0.0291 *	0.00261	0.0207	0.0335 **	0.0455 ***	0.0352 ***
	(0.0165)	(0.0160)	(0.0145)	(0.0131)	(0.0143)	(0.0121)
Age	0.0134	0.0132	0.0144	0.00639	0.0143	0.0223 ***
	(0.0111)	(0.0107)	(0.00974)	(0.00877)	(0.00957)	(0.00810)
Boy	−0.0291	−0.0389	−0.0827 **	−0.00314	0.00962	−0.0557 **
	(0.0387)	(0.0374)	(0.0340)	(0.0307)	(0.0335)	(0.0283)
Urban	0.0536	−0.00668	0.00891	0.0522	0.0578*	0.0623 **
	(0.0404)	(0.0391)	(0.0355)	(0.0320)	(0.0349)	(0.0295)
Constant	3.788 ***	4.172 ***	4.184 ***	4.302 ***	3.818***	4.147 ***
	(0.195)	(0.189)	(0.172)	(0.155)	(0.169)	(0.143)
Observations	2407	2407	2408	2408	2407	2406
*R* ^2^	0.004	0.001	0.005	0.005	0.008	0.012

Standard errors are given in parentheses; *** *p* < 0.01, ** *p* < 0.05, * *p* < 0.1.

## References

[1] State Council (2016). Notices on Strengthening the Care and Protection for Rural Left-Behind Children.

[2] Elgar F.J., Gariepy G., Torsheim T., Currie C. (2016). Early-life income inequality and adolescent health and well-being. Soc. Sci. Med..

[3] Bradshaw J., Hoelscher P., Richardson D. (2006). An Index of Child Well-being in the European Union. Soc. Indic. Res..

[4] EU Social Protection Committee (2008). Child Poverty and Well-Being in the EU: Current Status and Way Forward.

[5] Lau M., Bradshaw J. (2010). Child Well-being in the Pacific Rim. Child. Indic. Res..

[6] Lau M.K.W., Li W. (2011). The extent of family and school social capital promoting positive subjective well-being among primary school children in Shenzhen, China. Child. Youth Serv. Rev..

[7] Minujin A., Nandy S. (2012). Global Child Poverty and Well-Being: Measurement, Concepts, Policy and Action.

[8] UNICEF (2013). Child. Well-Being in Rich Countries: A Comparative Overview.

[9] Camfield L., Streuli N., Woodhead M. (2008). Children’s Well-Being in Contexts of Poverty: Approaches to Research, Monitoring and Participation.

[10] Chzhen Y., de Neubourg C. (2014). Multiple Overlapping Deprivation Analysis for the European Union (EU-MODA).

[11] Chzhen Y., de Neubourg C., Plavgo I., de Milliano M. (2014). Understanding Child Deprivation in the European Union: The Multiple Overlapping Deprivation Analysis (EU-MODA) Approach.

[12] De Milliano M., Plavgo I. (2014). Analysing child poverty and deprivation in sub-Saharan Africa: CC-MODA-Cross Country Multiple Overlapping Deprivation Analysis. Innocenti Work. Pap..

[13] De Neubourg C., Bradshaw J., Chzhen Y., Main G., Martorano B., Menchini L. (2012). Child Deprivation, Multidimensional Poverty and Monetary Poverty in Europe. Innocenti Work. Pap..

[14] De Neubourg C., Cai J.Q., De Milliano M., Plavgo I., Wei Z.R. (2012). Cross-Country MODA Study: Multiple Overlapping Deprivation Analysis (MODA).

[15] Gordon D., Nandy S., Minujin A., Nandy S. (2012). Measuring child poverty and deprivation. Global Child Poverty and Well-Being: Measurement, Concepts, Policy and Action.

[16] Notten G., Roelen K. (2011). Monitoring Child Well-Being in the European Union: Measuring Cumulative Deprivation.

[17] Qi D., Wu Y. (2014). Child Poverty in China-A Multidimensional Deprivation Approach. Child Indic. Res..

[18] Qi D., Wu Y. (2015). A multidimensional child poverty index in China. Child. Youth Serv. Rev..

[19] UNICEF (2011). Child Poverty in East Asia and the Pacific: Deprivations and Disparities.

[20] Diener E. (2006). Guidelines for National Indicators of Subjective Well-Being and Ill-Being. J. Happiness Stud..

[21] Boyden J., Dercon S. (2012). Child Development and Economic Development: Lessons and Future Challenges.

[22] Duncan G.J., Brooks-Gunn J. (1997). Consequences of Growing up Poor.

[23] Duncan G.J., Dunifon R., Doran M.W., Yeung W.-J.J. How Different are Welfare Families from Low-Income and Working Families? And Do Those Differences Matter for Children’s Achievement?. Proceedings of the Conference of the Family Process and Child Development in Low Income Families.

[24] Duncan G.J., Brooks-Gunn J., Klebanov P.K. (1994). Economic deprivation and early childhood development. Child. Dev..

[25] Fotso J.C., Madise N., Baschieri A., Cleland J., Zulu E., Mutua M.K., Essendi H. (2012). Child growth in urban deprived settings: Does household poverty status matter? At which stage of child development?. Health Place.

[26] Hannum E., Liu J., Frongillo E.A. (2014). Poverty, food insecurity and nutritional deprivation in rural China: Implications for children’s literacy achievement. Int. J. Educ. Dev..

[27] Kendall G.E., Li J. (2005). Early childhood socialization and social gradients in adult health: A commentary on Singh-Manoux and Marmot’s “Role of socialization in explaining social inequalities in health” (60: 9, 2005, 2129–2133). Soc. Sci. Med..

[28] Brooks-Gunn J., Duncan G.J. (1997). The Effects of Poverty on Children. Futur. Child..

[29] Patterson G., De Barsyshe B., Ramsey E. (1989). A developmental perspective on antisocial behavior. Am. Psychol..

[30] Conger K.J., Rueter M.A., Conger R.D., Crockett L.J., Silbereisen R.J. (2000). The role of economic pressure in the lives of parents and their adolescents: The family stress model. Negotiating Adolescence in Times of Social Change.

[31] Conger R.D., Wallace L.E., Sun Y., Simons R.L., McLoyd V.C., Brody G.H. (2002). Economic pressure in African American families: A replication and extension of the family stress model. Dev. Psychol..

[32] Horwitz S.M., Briggs-Gowan M.J., Storger-Isser A., Carter A.S. (2007). Prevalence, correlates and persistence of maternal depression. J. Women Health.

[33] Beck C.T. (1996). A Meta-Analysis of the Relationship Between Postpartum Depression and Infant Temperament. Nurs. Res..

[34] Brennan P.A., Hammen C., Anderson M.J., Bor W., Najman J.M., Williams G.M. (2000). Chronicity, severity, and timing of maternal depressive symptoms: Relationships with child outcomes at age five. Dev. Psychol..

[35] Brennan P.A., Hammen C., Katz A.R., Le Brocque R.M. (2002). Maternal depression, paternal psychopathology, and adolescent diagnostic outcomes. J. Consult. Clin. Psychol..

[36] Brennan P.A., Le Brocque R., Hammen C. (2003). Maternal Depression, Parent-Child Relationships, and Resilient Outcomes in Adolescence. J. Am. Acad. Child Adolesc. Psychiatry.

[37] Campbell S.B., Brownell C.A., Hungerford A., Spieker S.J., Mohan R., Blessing J.S. (2004). The course of maternal depressive symptoms and maternal sensitivity as predictors of attachment security at 36 months. Dev. Psychopathol..

[38] Fergusson D.M., Horwood L.J., Lynskey M.T. (1995). Maternal Depressive Symptoms and Depressive Symptoms in Adolescents. J. Child Psychol. Psychiatry.

[39] Halligan S.L., Murray L., Martins C., Cooper P.J. (2007). Maternal depression and psychiatric outcomes in adolescent offspring: A 13-year longitudinal study. J. Affect. Disord..

[40] Lim J., Wood B.L., Miller B.D. (2008). Maternal depression and parenting in relation to child internalizing symptoms and asthma disease activity. J. Fam. Psychol..

[41] Stewart R. (2007). Maternal depression and infant growth? A review of recent evidence. Matern. Child Nutr..

[42] Talge N.M., Neal C., Glover V. (2007). Early Stress, Translational Research and Prevention Science Network: Fetal and Neonatal Experience on Child and Adolescent Mental Health. Antenatal maternal stress and long-term effects on child neurodevelopment: How and why?. J. Child. Psychol. Psychiatry..

[43] O’Donnell K.J., Glover V., Barker E.D., O’Connor T.G. (2014). The persisting effect of maternal mood in pregnancy on childhood psychopathology. Dev. Psychopathol..

[44] Fletcher R.J., Feeman E., Garfield C., Vimpani G. (2011). The effects of early paternal depression on children’s development. Med. J. Aust..

[45] Kvalevaag A.L., Ramchandani P.G., Hove O., Assmus J., Eberhard-Gran M., Biringer E. (2013). Paternal Mental Health and Socioemotional and Behavioral Development in Their Children. Pediatrics.

[46] Wilson S., Durbin C.E. (2010). Effects of paternal depression on fathers’ parenting behaviors: A meta-analytic review. Clin. Psychol. Rev..

[47] Quinn A., Briggs H.E., Miller K.M., Orellana E.R. (2014). Social and familial determinants of health: Mediating effects of caregiver mental and physical health on children’s mental health. Child. Youth Serv. Rev..

[48] Diener E., Ng W., Harter J., Arora R. (2010). Wealth and happiness across the world: Material prosperity predicts life evaluation, whereas psychosocial prosperity predicts positive feeling. J. Pers. Soc. Psychol..

[49] Plouffe R.A., Tremblay P.F. (2017). The relationship between income and life satisfaction: Does religiosity play a role?. Pers. Individ. Differ..

[50] Lin K. (2014). Social Quality and Happiness—An Analysis of the Survey Data from Three Chinese Cities. Appl. Res. Qual. Life.

[51] Ren Q., Treiman D. (2016). The consequences of parental labor migration in China for children’s emotional well-being. Soc. Sci. Res..

[52] UNCRC (1989). Convention on the Rights of the Child.

[53] UNICEF (2007). Child Poverty in Perspective: An Overview of Child Well-Being in Rich Countries.

[54] Seligson J.L., Huebner E.S., Valois R.F. (2003). Preliminary Validation of the Brief Multidimensional Students’ Life Satisfaction Scale (BMSLSS). Soc. Indic. Res..

[55] Abedi M.R., Vostanis P. (2010). Evaluation of quality of life therapy for parents of children with obsessive–compulsive disorders in Iran. Eur. Child Adolesc. Psychiatry.

[56] Siyez D.M., Kaya A. (2007). Validity and Reliability of the Brief Multidimensional Students’ Life Satisfaction Scale with Turkish Children. J. Psychoeduc. Assess..

[57] Huebner E.S. (1991). Initial Development of the Student’s Life Satisfaction Scale. Sch. Psychol. Int..

[58] Huebner E.S. (1994). Preliminary development and validation of a multidimensional life satisfaction scale for children. Psychol. Assess..

[59] Adelman H.S., Taylor L., Nelson P. (1989). Minors’ dissatisfaction with their life circumstances. Child Psychiatry Hum. Dev..

[60] Goodman R. (1997). The Strengths and Difficulties Questionnaire: A Research Note. J. Child Psychol. Psychiatry.

